# Metabotropic Glutamate Receptor 5 Negative Modulation in Phase I Clinical Trial: Potential Impact of Circadian Rhythm on the Neuropsychiatric Adverse Reactions—Do Hallucinations Matter?

**DOI:** 10.1155/2014/652750

**Published:** 2014-03-04

**Authors:** Khalid Abou Farha, Richard Bruggeman, Corine Baljé-Volkers

**Affiliations:** ^1^Rob Giel Research Centre, Department of Psychiatry, University Medical Centre Groningen (UMCG), Hanzeplein 1, 9713 GZ Groningen, The Netherlands; ^2^Department of Epidemiology (Medical Statistics), University Medical Centre Groningen (UMCG), Hanzeplein 1, 9713 GZ Groningen, The Netherlands

## Abstract

Metabotropic Glutamate Receptor 5 (mGluR5) negative allosteric modulators (NAMs) may play a role in some psychiatric disorders such as anxiety and depression. The pharmacokinetic profile and pharmacodynamics effects of mGluR5-NAMs have been previously reported. We performed a post hoc analysis of pharmacological and clinical data obtained from 18 young healthy female subjects who received a mGluR5-NAM in the context of a phase I drug-drug interaction study between a mGluR5 NAM and a monophasic oral contraceptive. mGluR5-NAM was administered in an escalating bidaily dose level design. There was no interaction between the OC and mGluR5-NAM. Higher morning mGluR5-NAM plasma concentrations were found compared to evening concentrations. Most of the observed clinically significant neuropsychiatric adverse reactions occurred nocturnally and included visual (pseudo) hallucinations, insomnia accompanied by secondary behavioural disorders, and cognitive dysfunction symptoms of sufficient severity to interfere with daily functioning. Circadian rhythm-related physiological variations in drug absorption and disposition may explain this pharmacokinetics-pharmacodynamics apparently disproportionate relationship. We suggest that clinical trials evaluating basic pharmacokinetic properties of psychiatric medications consider potential drug's chronopharmacokinetics. This may assist with dose optimization and minimize serious neuropsychiatric adverse reactions in the vulnerable psychiatric patient.

## 1. Introduction

Metabotropic glutamate receptors, members of the G protein-coupled receptor superfamily, are widely distributed throughout the central nervous system (CNS) on neuronal and glial cells. During the past two decades, a body of scientific evidence has accumulated indicating that metabotropic glutamate receptors play a substantial role in many CNS diseases and psychiatric disorders [1, 2]. Given their discrete localization within the human brain and thence the possibility of limiting off-target effect, metabotropic glutamate receptors subtype 5 (mGluR5) have been proposed as an attractive novel therapeutic target worth investigating [2]. In this context, negative allosteric modulation of mGluR5 has been suggested for the treatment of mental disorders, such as anxiety disorders and depression, and more recently for treatment of alcohol and drug addiction [1, 2]. A number of mGluR5 antagonists and selective NAMs have been identified in the last decade [1]. Selective mGluR5-NAMs have been reported to possess higher potency, selectivity, and brain penetrance and demonstrate a better safety profile compared to mGluR5 competitive antagonists. However, relevant mGluR5-NAM-related adverse effects including cognitive and memory impairments and psychotomimetic effects have also been reported [1, 3].The exact underlying mechanism and implication of mGluR5 in the pathogenesis of these adverse effects remain to be verified. An overview of studies evaluating mGluR5 in preclinical and clinical settings is provided in the review papers of Gregory et al. [1] and Durand et al. [4].

Here we report our clinical and pharmacological experience with bidaily ascending oral doses of mGluR5-NAM administered to a cohort of 18 young healthy female volunteers. The medication used in this trial, a mGluR5-NAM, is a structurally novel lipophilic compound with a Log *P* value of 4.7. In previous exploratory pharmacokinetic assessments of this medication (data not published), the following drug characteristics were obtained: time to maximum exposure (*t*_max⁡_): 2 h, half-life (*t*_1/2_): 7 h and plasma albumin binding: >97%. A positive food effect on oral bioavailability particularly *C*_max⁡_ was found. The test drug is a noninhibitor, noninducer substrate of CYP 2C, CYP 1A1, and CYP3A4. It is not a substrate of intestinal or hepatic active efflux transport processes. The test drug major metabolites are all inactive. Elimination of the parent drug and its metabolite occur mainly via the faecal route. In faeces, ca. 41% of the test drug dose is eliminated in an unchanged form.

## 2. Subjects and Methods

A cohort of 23 healthy nonsmoking female subjects aged between 18 and 37 years with a mean age of 24.2 years were recruited to participate in a phase I clinical trial to evaluate the potential pharmacokinetic interaction between a novel mGluR5-NAM, test drug, and a monophasic oral contraceptive (OC). The current report demonstrates a subanalysis of pooled data from 18 female subjects enrolled in a phase I drug-OC interaction clinical trial. The clinical trial protocol, related amendments, and informed consent including statement of willingness to participate in the clinical study (English and Dutch) were reviewed (from legal, ethical, and medical points of view) and approved by the independent Ethics Committee, of the “Evaluation of Ethics in Biomedical Research” (BEBO) Foundation, Assen, The Netherlands.

The medical history (as indicated by subjects own reports and confirmed by subjects general practitioners) including history of psychiatric diseases, alcohol and drug abuse, physical examinations, thorough laboratory investigations, and ECG assessments indicated the mental and physical healthy, drug nonaddiction states of all participants. All subjects had negative pregnancy and drug screening tests at screening and at admission. No positive family history of mood disorders was obtained from any of the study participants. Written informed consent was obtained from all 23 subjects before initiation of any study-related procedure. The study was conducted in an inpatient setting under strictly controlled conditions. Data obtained from five of the 23 consented subjects has not been included in the statistical analysis of this report. Two subjects were prematurely removed on the second and third days after starting the study (during dose up-titration period), having demonstrated severe drug-induced hallucinations during the first and second dosing days. Both subjects did not complete the study, and therefore, data required for statistical analysis were not collected/unavailable. One subject withdrew her consent due to personal reason. One subject developed urinary tract infection and was removed from the study before receiving the study medication. One subject became pregnant (has had positive pregnancy test) at the end of the study. The statistical models were run with and without the data from this subject, only to check if the data of this subject would influence the outcome. Although subject's data were not considered clinically (see Section 3) and/or statistically different from the other subjects, data were excluded from the report to avoid any pregnancy-induced pharmacodynamic or pharmacokinetic confounding effect.

The test medication was administered orally (capsule formulation) in an escalating bidaily dose level design including an up-titration (dose-escalation) period of 3 days followed by a 9-day fixed-dose (steady state) period in which the same dose level of the test drug was orally administered twice a day (as 2 equal doses, 12 hours apart, between 08:00 and 08:30 and 20:00 and 20:30). No placebo was administered in this study. During the up-titration period and throughout the fixed-dose (steady state) period, with the exception of Day 7, the morning dose was administered half an hour before breakfast. On Day 7 of the steady state, the test drug was administered under fasting conditions. On all days of the clinical trial a standard meal was given 2 h before evening dose. From 2 weeks prior to the first dosing and throughout the study, use of medications or consumption of food or drinks known to influence the CYP 450 enzyme activity, for example, grape fruit, was not allowed.

Blood samples (3 mL) were obtained from participating subjects, at different time points of the study, to assess drug concentrations. During the 3 up-titration days and on Days 1 and 7 of the fixed-dose period, blood samples were obtained at the following time points: before morning dose (0 h), 2 hours after morning dose and 2 hours after evening dose. On Day 7 of the fixed-dose period 2 additional blood samples were taken: one sample before evening dose (0 h) and one sample 4 hours after evening drug administration. On Day 1 of the fixed-dose (steady state) period, the evening test drug concentration at 0 h was not assessed. On all other days of the fixed-dose period test drug serum concentration was assessed at one time point, 2 hours after morning dose.

The blood samples were collected in tubes containing sodium heparin anticoagulant and centrifuged. The tubes were kept frozen at below −20°C until time of analysis. The test drug concentrations were determined in plasma by a validated liquid chromatography-mass spectrometry/mass spectrometry (LC-MS/MS) method with a lower limit of quantitation (LLOQ) of 2 ng/mL.

Throughout the study the subjects were closely monitored and queried about the occurrence of subjective complaints at regular intervals using nonleading questions. Any subjective complaint or objective finding encountered during the study was reported by the medical staff in study specific medical (nursing) notes. All reported or observed adverse events were assessed and evaluated by the principal investigator of the study. The most relevant CNS adverse events encountered in this trial were insomnia, anxiety and emotional lability, cognitive impairment symptoms, and visual (pseudo) hallucination. The latter was defined according to the DSM IV criteria [5] as a sensory perception without external stimulation of the relevant sensory organ. In this report visual (pseudo) hallucinations have been classified into simple hallucinations, characterised by absence of forms and are often photopsias such as flashes of light or colours and complex hallucinations, having specific forms that might include animals, objects, and humans [6, 7].

*Statistical Analysis.* We performed a post hoc analysis of the results obtained from 18 healthy young female volunteers with regard to the PK profile and the drug-related adverse reaction. The concentration data were statistically analyzed using SAS version 9.1.3. A mixed model was used for comparison of morning and evening 2 h postdose concentrations for Days 1 and 7 of the steady state. The amount of drug absorbed and remained in the circulation at 2 hours postmorning and -evening dosage on Day 7 of the fixed-dose (steady state) period was calculated by subtracting *C*_trough_ (trough plasma drug concentration obtained just before dosing at 0 h) from *C*_max⁡_ (concentration at 2 h after dose). An ANCOVA was used to compare the difference between 2 h postdose and baseline trough (0 h) values obtained from morning and evening concentrations on Day 7, with the baseline trough value as additional covariate. In both models, contrasts were defined to obtain the results for subgroup comparisons. The evening plasma concentrations obtained at 2 h and 4 h after dose on Day 7 were compared using a paired *t*-test. All found *P* values are two-sided, and alpha = 0.05 has been used. No correction for multiple comparisons was done, as these post hoc results are considered explorative and the main aim is to identify hypotheses that should be subjected to further research.

## 3. Results

**Figure 1:**
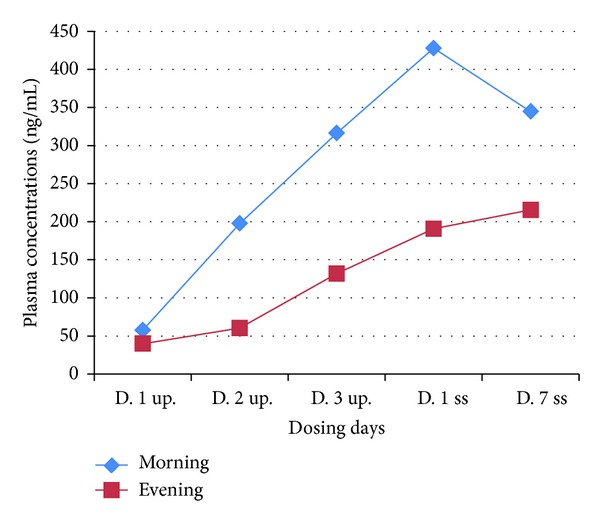
Mean plasma concentrations (ng/mL) obtained 2 hours after morning and evening dosage, during the up-titration phase and Days 1 and 7 of the steady state period. UPT = up-titration phase; SS = steady state period.

**Figure 2:**
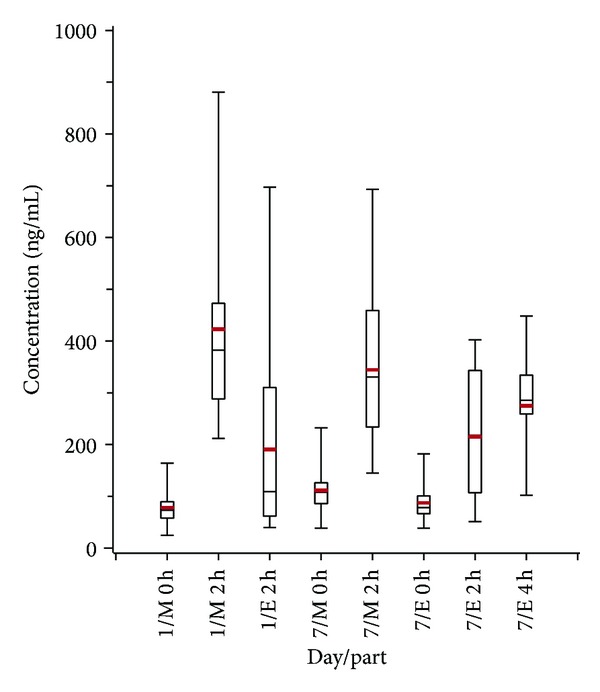
Boxplot of the data values obtained on Days 1 and 7 of steady state period; M = morning, E = evening.

**Figure 3:**
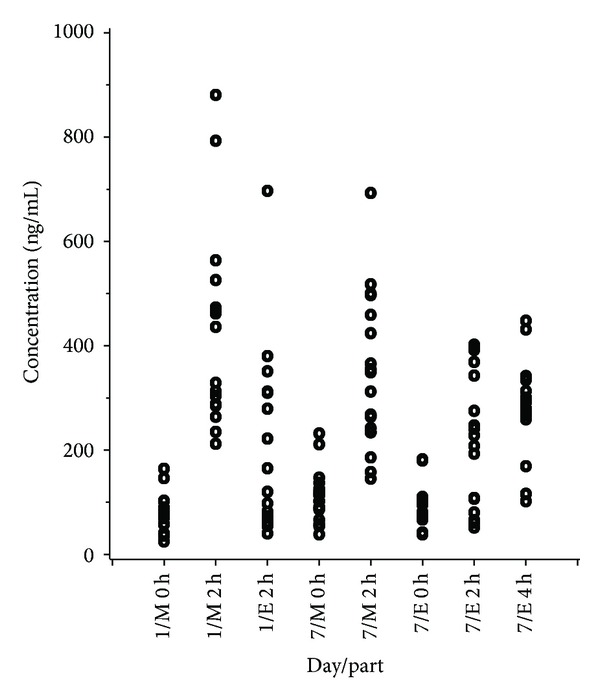
Individual data values obtained on Days 1 and 7 of steady state; M = morning, E = evening.

On the 3 up-titration days and on Days 1 and 7 of the fixed-dose (steady state) period the mean plasma levels obtained 2 h after morning dose were consistently higher than those obtained 2 h after evening dose (Figure 1). For both Day 1 and Day 7, 15 out of 18 subjects (83%) showed a smaller evening concentration. A comparison of the results from Day 1 and Day 7 showed an overall statistically significant difference between morning and evening concentrations (*P* < 0.0001), Figures 2 and 3. This difference was also seen when each day was separately analyzed but was less pronounced for Day 7 (*P* < 0.0001 for Day 1; *P* = 0.002 for Day 7), Tables 1 and 2.

**Figure 4:**
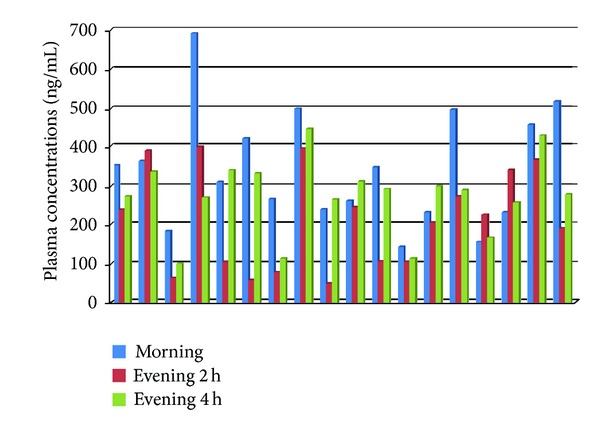
Day 7 of steady state period, individual plasma concentrations (ng/mL), morning at 2 h postdose and evening at 2 h and 4 h postdose time points.

Based on the descriptive statistics, it was apparent that a difference existed between the morning and evening baseline values. This difference proved to be statistically significant (*P* = 0.0292) and was, therefore, included in the ANCOVA model as an additional covariate. In the ANCOVA for the Day 7, difference in plasma concentrations between 2 h and 0 h, a statistically significant difference was found between morning and evening concentrations, Table 2 (*P* = 0.0165). We also compared the evening test drug plasma concentrations obtained on Day 7 at 2 h with those obtained at 4 h after dosage. The mean of concentrations obtained at 4 h after evening dose was 28% higher than that obtained 2 h after evening dosage (275 ng/mL versus 215 ng/mL). This difference was statistically significant (*P* = 0.0357). At the individual subject level, the 4 h postevening plasma concentrations were higher than those obtained at 2 h after dose in 14 out of the 18 subjects (78%); see Figure 4.

In terms of the adverse events (clinical PD effects), an array of CNS-related symptoms was encountered in all participating subjects. These ranged from mild episodes of headache, light headedness, and fatigue to symptoms of cognitive dysfunction, anxiety, insomnia, and (pseudo) hallucinations. Twenty-three (23) visual (pseudo) hallucinatory episodes were reported in 11 out of 18 (61%) subjects, that is, not including 3 subjects who also demonstrated (pseudo) hallucination at some point during the trial: 2 prematurely withdrawn subjects with severe hallucination and one subject who became pregnant at the end of the study. The (pseudo) hallucinatory episodes were nocturnal in 9 out of the 11 subjects (82%), diurnal in one subject (9%), and combined diurnal-nocturnal in one subject (9%). In descriptive terms, simple (pseudo) hallucinatory episodes consisted mainly of flashes of light. The latter were reversible and not accompanied by migraine or associated with vision loss or other ophthalmic disorders. None of the subjects had a history or clinical symptoms or signs of any eye disease at screening, during the conduct of study or at the follow-up visit.

The content of complex (pseudo) hallucinatory episodes included distorted baby faces, animal faces, spiders with yellow legs and red mosquitos, mythical figures, seeing people from the distant past (who were not physically present), and moving dolls. The encountered (pseudo) hallucinations were nocturnal complex in 8 subjects (recurrent in 1 subject); diurnal complex (1 episode) in one subject; one nocturnal complex and 1 diurnal simple (pseudo) hallucinatory episode in one subject; and 11 nocturnal (pseudo) hallucinatory episodes (4 complex and 7 simple) in one subject. In all of the hallucinatory subjects, visual (pseudo) hallucination occurred without any precipitating factor while they were awake. Hallucinations were of sudden onset, occurred 45 minutes to 5 hours and 20 minutes after dose, and lasted for 5 minutes to 12 h (Table 3). It is also worth mentioning that, in 2 subjects (data not included in the analysis), the test drug was prematurely discontinued due to severe nocturnal combined visual complex and tactile (pseudo) hallucinatory episodes. One of these 2 subjects saw dust whirling from the ceiling and experienced electricity cable wiring on her body. The hallucination was so severe that the subject tried to protect her own belongings from the dust by covering them with clothing and thereafter started to clean the hallucinatory dust from her own arms and legs. This nocturnal hallucinatory episode persisted for 7 hours and was followed by emotional lability and severe impairment of cognitive functions (mental absence) that lasted for a further 6.5 hours. In the case of the other prematurely withdrawn subjects, the (pseudo) hallucinatory episode also contained 2 components, a visual and a tactile component. The visual hallucinatory component consisted of mixed simple and complex visual hallucinations. The subject saw purple, yellow, and green-colored flashes of light together with water drops and flowers. The tactile component consisted of the feeling that the bed was folding (up) around her body. This nocturnal combined visual and tactile hallucinatory episode lasted for almost 2 hours and was combined with anxiety. The next morning the subject remained restless and demonstrated difficulty with expressing her thoughts. This was associated with intense concentration impairment and mental dullness which lasted for 14.5 continuous hours. As mentioned above, the data of one subject who became pregnant at the end of the study was not included in the analysis in order to avoid any pregnancy-induced confounding effect. This subject demonstrated one diurnal and 2 nocturnal episodes of complex (pseudo) visual hallucination. All 3 episodes occurred during the fixed-dose period of the study. The diurnal episode started ca. 20 minutes after the morning dose and lasted for 1 hour and 20 minutes. This hallucinatory episode was associated with restlessness and consisted of seeing images (described as movie scenes) from old job situations. The second episode, a nocturnal one, consisted of hypnagogic frightening images (as described by the subject) that occurred at the boundary between sleeping and waking. This episode started 4 hours and 50 minutes after the evening dose and continued for 1 hour and 50 minutes after the subject woke up from a sleep. The third (pseudo) visual hallucinatory episode started 3 hours and 55 minutes after the evening dose and lasted for 1 hour and 15 minutes. The subject saw a ceiling fan falling down together with continuously changing figures. This was frightening for the subject and necessitated close medical supervision and assistance, by supervisory physician, to calm down the subject. Noteworthy is that the ward on which the subject was admitted has no ceiling fan.

Nocturnal insomnia associated with significant secondary behavioural disorders including anxiety and emotional instability was observed in 13 out of the 18 participating subjects (72%). Ten of these 13 subjects (ca. 77%) also experienced visual (pseudo) hallucinations. Symptoms of cognitive dysfunction of sufficient severity to interfere with daily functioning were encountered in 11 of the 18 subjects (61%). Seven of these 11 subjects (ca. 64%) also experienced visual (pseudo) hallucinations. The cognitive dysfunction symptoms experienced were impaired concentration in 8 subjects (duration range 0.5–13.75 h), mental dullness in 5 subjects (lasting from 3.75 to 72 h), slow reaction to sensory stimuli including delayed visual perception in 5 subjects (duration range 1–35 h), and difficulty expressing thoughts in 2 subjects (lasting for 1.5 and 45 h, resp.).

## 4. Discussion

In this post hoc analysis, we studied the morning and evening peak plasma concentrations (*C*_max⁡_) and the associated adverse events of a twice-daily oral administration of mGlu5R-NAM, a class of compounds suggested for the treatment of some psychiatric disorders such as anxiety and depression. The data demonstrated in this report raises 2 points of pharmacological as well as clinical interest. First, we observed intrasubject differences between morning and evening peak plasma concentrations (*C*_max⁡_) at 2 h after dosing of the test drug in 2 equal dose levels. In this respect, it is noteworthy to mention that some critical pharmacokinetic properties of medicinal drugs such as *t*_max⁡_, *C*_max⁡_ and *t*_1/2_ are usually based on morning dose studies. Extrapolation of these basic PK properties from a diurnal drug plasma concentration-time curve alone might underestimate the clinical relevance of drug chronopharmacokinetics. The reported data shows that the mean of 2 h postdose evening plasma concentrations obtained on Day 1 and Day 7 of the fixed-dose (steady state) period was, respectively, 45% (191/422 ng/mL) and 62% (215/344 ng/mL) of the corresponding morning mean concentration. On Day 1, the morning dose was administered half an hour before breakfast, while, on Day 7, the morning dose was administered under fasting conditions. A food effect on the test drug bioavailability could explain the difference between the morning concentrations on Days 1 and 7. However, the difference between the morning and evening concentrations on Day 7 more likely indicates the influence of endogenous intrasubject factors that control drug absorption and disposition (distribution and elimination by biotransformation and excretion). This assumption is supported by looking at the average difference between *C*_max⁡_ and *C*_trough_ in the evening of Day 7, which was found to be 55% of the corresponding average difference between *C*_max⁡_ and *C*_trough_ in the morning (128.5/233.0 ng/mL).

The apparent low evening *C*_max⁡_ of this lipophilic drug might be explained by the impact of endogenous biological rhythm on factors controlling drug absorption and disposition. The impact of circadian rhythm on drug plasma therapeutic levels has been discussed in the reports of Sukumaran and coworkers [9] and Erkekoglu and Bayder [10]. Circadian rhythm induces an increase in evening gastric emptying time, intestinal hypomotility, low hepatic and gastrointestinal blood flow, and decreased expression of intestinal lipophilic drug transporters genes such as microsomal triglyceride transfer protein, apolipoproteins B, and apolipoprotein A4 [9, 10]. As mentioned above, approximately 41% of the test drug is eliminated unchanged in faeces. Intestinal hypomotility leads to prolonged contact of the test drug with the gut epithelium, and therefore, an increase, rather than decrease, in the amount of drug absorbed would be expected. Taken together, this suggests a delay in reaching evening peak plasma concentrations, which in turn might give a false conclusion of a low *C*_max⁡_ value when assessed at the same postdose time point used for assessment of the morning *t*_max⁡_. The data demonstrated in this report strengthen this assumption. On Day 7, the evening test drug concentrations taken at 4 h after dose were higher than those taken at 2 h after dose in 78% of participating subjects. Also the mean of individual plasma concentrations obtained at 4 h after evening dose was 28% higher than that obtained 2 h after evening dose (275 ng/mL versus 215 mg/mL). Given that, it might be conceivable to extrapolate the critical PK properties of lipophilic medicinal products (such as those used to treat CNS diseases and psychiatric disorders) not only from diurnal drug plasma concentration-time curves but also from evening ones. Considering evening/nocturnal pharmacokinetic studies in addition to the conventional morning pharmacokinetic studies might reveal drugs chronopharmacokinetics and allow a better understanding of human body-drug interaction.

Second, the rate of clinically relevant drug-induced neuropsychiatric adverse reactions, such as complex visual (pseudo) hallucinatory episodes and complicated insomnia was higher after evening dose. The exact underlying mechanism of this apparent discrepancies between prevailing plasma concentrations and CNS effects is not clear. Hysteresis caused by delayed secondary pharmacological effects of mGluR5 inhibition such as diminished activation of N-methyl-D-aspartate receptors (NMDARs) offers no likely explanation. This is because the drug was administered twice daily, meaning that, if delayed pharmacological effect is the cause, then the CNS adverse effects would have been encountered with similar frequencies during the day and at night. A possible explanation is nocturnal exposure of the brain to a higher concentration of the test drug. Circadian rhythm-related physiological variations in hepatic and extrahepatic drug cytochrome enzyme activity, hepatic blood flow (HBF), serum albumin levels, and cerebral blood flow (CBF) may play a role in this scenario. The test drug is metabolized by CYP 450 enzymes 2C, 1A1, and 3A4 which are present not only in the liver and gut, but also in the brain [11–13]. A large number of CYP 450 enzymes including 2C, 1A1, and 3A4 are expressed by endothelial cells of human brain microvessels, blood brain barrier, and in approximately 75% of brain neurons. They are found concentrated near drug targets in specific regions [12, 13]. The levels of CYPs in specific neurons may be comparable to, or even higher than, levels in hepatocytes [13]. Brain CYPs might, therefore, exhibit a second pass effect and exert a relevant impact on local drug metabolism. The activity of these microsomal enzymes shows circadian rhythm that is controlled by the central body clock. The latter is regulated by light/dark phases and not by activity state [14]. In this context, mRNA expression of phase 1 detoxification enzyme system (microsomal cytochrome P450) and drug metabolism, via cytochrome P450 system, were reported to reach their peak during the day (active phase) when compared to night, resting phase [9, 10, 14]. With regard to HBF, Lemmer and Nold [15] found significant circadian phase-dependent variation in estimated HBF in healthy subjects, being greatest at 08:00 AM. Given the fact that both CYP 450 activity and HBF are major determinant factors in the rate of clearance of lipophilic drugs, circadian rhythm-related changes in these factors could respectively lead to a morning increase and evening decrease in the rate of mGlu5R-NAM biotransformation to its inactive metabolite. Regarding the circadian rhythm-induced alteration in serum albumin concentrations, Touitou et al. [16] reported that human plasma albumin concentrations drop to their lowest level during the night time and increase by day reaching a peak at around 08:00 AM, shortly after waking (circadian amplitude 8–13%). Although plasma albumin level has not been assessed in this study, circadian variation in plasma protein level might have influenced the ratio of test drug bound to unbound fraction (fu) in the plasma, causing nocturnal increase in plasma concentration of the mGluR5-NAM fu. The latter is the pharmacologically active form of mGlu5R-NAM. Lastly, it is widely agreed upon that CBF is lower in the morning shortly after awakening than in the afternoon or evening, nadir around 12:00 PM and a zenith at approximately midnight [17, 18]. Taken all together, nocturnal decrease in drug biotransformation to inactive metabolite and increases in drug fu and CBF may have caused higher nocturnal mGluR5-NAM concentration in the brain.

Our results show three important PD effects which may have implications in the psychiatric clinical settings. These effects are insomnia (with secondary mood disturbances including anxiety and emotional instability), visual (pseudo) hallucination, and symptoms of cognitive dysfunction of sufficient severity to interfere with daily functioning. Insomnia may be explained by a mGluR5-induced indirect activation of the hypothalamus-pituitary-adrenal (H-P-A) axis. The activity of HPA axis is under robust GABAergic inhibitory control [19]. GABAergic interneurons contain mGluR5 [20]. Inhibition of mGluR5 has been reported to induce decrease in inhibitory drive from GABAergic interneurons on HPA axis neurons [4, 21]. This ultimately leads to activation of the stress hormone axis with increased ACTH and cortisol release [4, 21], causing hyperarousal and insomnia [4, 22]. From a clinical point of view, treatment-induced activation of stress reaction and insomnia are crucial side effects that might have significant implication on psychiatric patients treated for anxiety disorder.

Although hyperarousal state and insomnia could explain some drug-induced visual hallucinations [6], the exact role of mGluR5 in the pathogenesis of hallucinations remains to be clarified. Another possible mechanism for the observed (pseudo) hallucination is N-methyl-D-aspartate receptors (NMDARs) hypofunction. Activation of NMDARs was found to increase hippocampal GABAergic inhibitory transmission [23], while noncompetitive inhibition of NMDARs induces cognitive impairment and psychotic symptoms such as hallucination [8]. mGluR5 receptors are physically coupled to NMDARs. Crosstalk with molecular and biochemical interactions between NMDARs and mGluR5 has been reported [24, 25].

Symptoms of cognitive dysfunction were demonstrated by 61% of the subjects in this report; 64% of these demonstrated visual (pseudo) hallucinations as well. In line with these findings, earlier reports indicated a relationship between mGluR5 antagonists/NAMs and cognitive impairment both in humans and animals [1, 3]. A link between cognitive impairment and complex visual hallucination in some CNS disease states has also been reported in the literature [7, 26, 27]. Regardless of the underling mechanism of mGlu5R associated (pseudo) visual hallucinations and cognitive dysfunction, attention should be given to the emergence of such clinically significant CNS adverse reactions in a mentally compromised psychiatric patient treated for depression or anxiety disorders.

In summary, this report sheds light on two important clinicopharmacological points. Firstly, critical pharmacokinetic properties of medicinal drugs such as *t*_max⁡_, *C*_max⁡_ and *t*_1/2_ are commonly determined from morning dose studies only. Extrapolation of basic PK properties from a diurnal plasma concentration-time curve may underestimate the clinical relevance of drug chronopharmacokinetics. We, therefore, suggest that clinical trials designed to evaluate critical PK characteristics of lipophilic medications targeting CNS diseases/psychiatric disorders consider potential chronopharmacokinetics of drugs. This might necessitate conducting PK studies not only in the morning but also in the evening. Secondly, CNS lipophilic medicinal products may achieve higher nocturnal brain drug concentration (compared to diurnal one) possibly due to circadian rhythm-related physiological variations in major determinants of drug disposition. This might result in emergence of more nocturnal neuropsychiatric adverse reactions. Further investigations are needed to confirm this observation and to elucidate its exact underlying mechanisms including possible circadian variation in plasma levels of drug unbound fraction.

## Figures and Tables

**Table 1:** Descriptive statistics, Day 1 and Day 7 concentrations (ng/mL), 2 h/4 h after dose.

Day, time point	Day part	Mean (SD)	Median	Min–max
1, 2 h after dose	Morning	422.4 (184.2)	382.5	212–881
1, 2 h after dose	Evening	190.8 (171.9)	108.9	40.0–697
7, 2 h after dose	Morning	344.7 (146.9)	330.5	145–693
7, 2 h after dose	Evening	215.3 (126.4)	218.0	51.3–402
7, 4 h after dose	Evening	274.9 (97.3)	285.5	201–448

**Table 2:** Descriptive statistics, Day 7 concentrations (ng/mL), 0 h and 2 h after dose.

Day part	Value	Mean (SD)	Median	Min–max
Morning	Baseline value (0 h)	111.4 (49.7)	107.5	38.4–232
	Concentration (2 h)	344.7 (146.9)	330.0	145–693
	Difference	233.3 (114.9)	214.0	91.0–546

Evening	Baseline value (0 h)	86.9 (41.1)	78.4	38.5–182
	Concentration (2 h)	215.3 (126.4)	218.0	51.3–402
	Difference	128.5 (109.8)	133.6	−16.0–313.5

**Table 3:** Onset and end of (pseudo) hallucinations reported from/observed in 11 subjects during the course of the clinical trial.

Subject number	Dosing times*	Number of episodes	Onset*	End*
2	08:05 and 20:05	1	C: 00:15	00:45

3	08:10 and 20:10	1	C: 01:30	02:00

5	08:20 and 20:20	1	C: 23:30	01:30

6	08:25 and 20:25	2	C: 01:07	02:00
			C: 01:30	08:00

8	08:00 and 20:00	1	C: 08:45	08:55

9	08:00 and 20:00	1	C: 23:00	23:30

10	08:05 and 20:05	1	C: 01:00	02:30

13	08:20 and 20:20	1	C: 00:30	00:35

15	08:15 and 20:15	3	S: 09:50	11:00
			C: 00:00	03:00

16	08:25 and 20:25	11	C: 02:00	03:00
			C: 00:00	07:30
			C: 00:00	02:30
			C: 23:30	08:00
			S: 22:00	08:00
			S: 21:30	03:30
			S: 21:00	09:00
			S: 21:15	07:29
			S: 21:30	08:00
			S: 21:25	07:30
			S: 21:30	23:00

17	08:30 and 20:30	1	C: 00:20	03:30

*Clock time (h:minutes); C: complex (pseudo) hallucination; S: simple (pseudo) hallucination. Insomnia: 11–19; 24–28.

## Limitation of the Study

A limitation of this report is that the data presented here was post hoc extraction from pharmacokinetic and medical reports of participants in a phase I clinical trial. Thus, verification of these unforeseen findings by means of extra PK assessments (including 4 h postmorning dose) or PD tests, such as evaluation of diurnal and nocturnal plasma albumin levels and plasma concentration of drug unbound fraction, or cognitive tests and HPA axis hormones, was not possible since the reported findings were unforeseen, and therefore, this type of data was not collected during the trial. Further investigations to confirm and explore the clinical significance of these observations are thus warranted.
